# Effects of Electrolyzed Functional Water Combined with Modified Atmosphere Storage on Postharvest Quality of Arrow Bamboo Shoots

**DOI:** 10.3390/foods15162899

**Published:** 2026-08-19

**Authors:** Yan Tang, Yuan Long, Dandan Yan, Wei Yu, Shanglian Hu, Bo Zhao

**Affiliations:** Engineering Research Center of Biomass Materials, Ministry of Education, College of Life Sciences and Agri-Forestry, Southwest University of Science and Technology, Mianyang 621010, China; yantang3477@163.com (Y.T.); yuanlongswust@163.com (Y.L.); yandandan3599@163.com (D.Y.); 13568893896@163.com (W.Y.); hnw57@ustc.edu.cn (S.H.)

**Keywords:** *Fargesia spathacea* Franch, electrolysis of water, lignification, metabolomics, microbial community

## Abstract

Postharvest lignification and microbial spoilage limit the shelf life of arrow bamboo shoots. This study evaluated the effects of electrolyzed functional water (EFW) combined with spontaneous modified-atmosphere (MA) packaging (EFW+MA) on postharvest quality and associated physiological, metabolic, and microbial changes. EFW+MA reduced weight loss, decay incidence, and browning while maintaining firmness and color. It also reduced lignin and cellulose accumulation, accompanied by lower activities of phenylalanine ammonia-lyase (PAL), peroxidase (POD), and polyphenol oxidase (PPO). Untargeted liquid chromatography–tandem mass spectrometry (LC-MS/MS) metabolomics showed that storage-associated metabolic changes were moderated by EFW+MA, including lower abundances of phenylalanine and tyrosine in the treated samples. In addition, EFW+MA enhanced superoxide dismutase (SOD), catalase (CAT), and ascorbate peroxidase (APX) activities, reduced malondialdehyde (MDA) accumulation, and alleviated membrane lipid peroxidation. 16S ribosomal RNA (16S rRNA) gene sequencing showed that EFW+MA reduced operational taxonomic unit (OTU) richness and altered bacterial community composition, including lower relative abundances of *Campylobacterota* and *Enterobacteriaceae* and a higher relative abundance of *Lactobacillaceae*. Overall, EFW+MA delayed postharvest quality deterioration of arrow bamboo shoots, accompanied by changes in lignification-related physiology, oxidative defense, metabolite profiles, and bacterial communities.

## 1. Introduction

Bamboo shoots are rich in dietary fiber, amino acids, proteins, and minerals and are considered a healthy food with high protein, high fiber, and low fat content. They are favored by consumers because of their tender texture and high nutritional value [1]. Arrow bamboo shoots (*Fargesia spathacea* Franch) are mainly distributed in Sichuan, Yunnan, and Tibet, China, and contain abundant dietary fiber, vitamins, and minerals such as potassium, magnesium, and calcium, giving them high nutritional value and potential for development and utilization. However, bamboo shoots still maintain vigorous respiratory metabolism after harvest, and mechanical damage during harvesting and transportation can easily promote microbial proliferation, triggering a series of physiological and biochemical reactions that lead to tissue senescence, decay, and quality deterioration [2]. Therefore, adopting appropriate preservation measures to inhibit postharvest physiological metabolism and microbial growth is of great significance for delaying quality deterioration in bamboo shoots.

Lignification is one of the major causes of postharvest quality deterioration in bamboo shoots. During storage, components such as lignin and cellulose continuously accumulate in the cell walls of bamboo shoots, leading to increased tissue firmness, poorer eating quality, and reduced commercial value [3]. Previous studies have shown that treatments such as low temperature and low-voltage electrostatic fields can delay lignification and maintain the storage quality of bamboo shoots by inhibiting the activities of enzymes related to lignin biosynthesis and regulating metabolite changes [4,5]. In addition to lignification, microbial contamination is also an important factor limiting the shelf life of fresh bamboo shoots. The surfaces and cut sections of bamboo shoots are susceptible to contamination by microorganisms such as *Escherichia coli*, *Salmonella*, and *Pseudomonas*, which further accelerates decay and spoilage. Non-thermal sterilization technologies, such as cold plasma and irradiation, have been shown to reduce microbial populations on bamboo shoot surfaces and maintain their postharvest quality to some extent [6,7].

At present, chitosan coating, natural antimicrobial agents, nitric oxide, melatonin, and MA have been widely used for postharvest preservation of fruits and vegetables, mainly by inhibiting respiratory metabolism, enzymatic browning, and tissue senescence [8,9,10]. However, these approaches may have limitations in practical application. For example, some treatments primarily regulate physiological metabolism but exhibit limited antimicrobial activity, whereas antimicrobial treatments alone may not sufficiently suppress respiration, lignification, and quality loss during storage. In addition, the use of certain chemical preservatives may raise concerns regarding residues, safety, cost, or consumer acceptance. Therefore, an effective and green preservation strategy that simultaneously controls microbial spoilage, respiratory metabolism, and lignification is required for fresh bamboo shoots.

EFW is a type of functional water generated by electrolysis and is characterized by specific pH, oxidation–reduction potential, and available chlorine concentration. It has attracted considerable attention because of its high sterilization efficiency, environmental friendliness, ease of application, and lack of harmful chemical residues and has been used for the washing, sterilization, and preservation of fruits and vegetables [11,12]. Nevertheless, EFW is mainly applied as a washing or sterilization treatment, and its antimicrobial effect may be transient; thus, EFW alone may not provide continuous regulation of respiration, water loss, lignification, and tissue senescence throughout storage. MA can regulate the proportions of oxygen (O_2_) and carbon dioxide (CO_2_) inside the package, thereby reducing respiration intensity and nutrient consumption, delaying tissue senescence, and suppressing the accumulation of cell wall components [13]. However, MA alone may not adequately reduce the initial microbial load on the surface and cut sections of bamboo shoots. Therefore, combining EFW with MA may provide complementary effects: EFW can reduce the initial microbial contamination, while MA can maintain a favorable in-package atmosphere during storage to suppress respiration, water loss, senescence, and lignification. This combined treatment is expected to provide an effective residue-free strategy for extending the cold-storage shelf life of bamboo shoots.

Our previous four-treatment study in Chinese thorny bamboo shoots (CK, EFW, MA, and EFW+MA) showed that the preservation performance generally followed the order EFW+MA > MA > EFW > CK [14]. At day 35, MA alone reduced respiration intensity by 29.04% relative to CK, compared with a 16.57% reduction with EFW alone; MA also reduced MDA accumulation by 18.76%, compared with 13.42% for EFW. In the same study, EFW+MA produced the strongest overall preservation effect, including the lowest respiration intensity and MDA accumulation and the most effective delay of quality deterioration and lignification. Chinese thorny bamboo shoots and arrow bamboo shoots are both small-diameter bamboo shoots belonging to the subfamily *Bambusoideae* (Poaceae) and share similar postharvest characteristics. Building on these findings, the present study investigated the physiological, metabolic, and microbial changes associated with EFW+MA treatment in arrow bamboo shoots. Physiological quality, lignification-related indicators, and bacterial-community changes during storage were systematically assessed, and untargeted metabolomics was used to characterize treatment-associated metabolic differences.

## 2. Materials and Methods

### 2.1. Materials and Treatments

The experimental material consisted of arrow bamboo shoots harvested from Qinglong Township, Yingjing County, Ya’an City, Sichuan Province, China. At harvest, shoots with uniform size and good appearance and without obvious mechanical damage, disease symptoms, or insect infestation were selected. The harvested shoots were transported to the laboratory within 3 h and pre-cooled at 8 °C for 4 h.

The EFW treatment conditions, including pH, available chlorine concentration, soaking duration, and polyethylene (PE) bag thickness, were selected based on our previous study [14]. Fresh arrow bamboo shoots were randomly divided into two groups: an untreated control group (CK) and an EFW+MA treatment group. For the EFW+MA treatment, bamboo shoots were immersed in electrolyzed functional water (pH 2.5; available chlorine concentration, 40 mg/L) for 20 min, naturally air-dried, and then packed in 45 μm thick PE preservation bags (10 shoots per bag). The PE bags were sealed without perforations. The CK samples received no treatment.

Each treatment comprised three independent biological replicates, with 60 bamboo shoots used per treatment. All samples were subsequently stored at 4 ± 1 °C for 35 d. Physiological and quality-related parameters were determined at 7 d intervals during storage (0, 7, 14, 21, 28, and 35 d). All physiological and quality-related measurements were conducted using three independent biological replicates per treatment at each sampling time (n = 3). Samples collected at 0 and 14 d were snap-frozen in liquid nitrogen and stored at −80 °C until untargeted metabolomic and microbial community structure analyses.

### 2.2. Gas Composition and Respiration Intensity

The O_2_ and CO_2_ concentrations inside the preservation bags were measured at regular intervals using a portable O_2_ meter (model CY-12CB; Jiande Meicheng Electrochemical Analysis Instrument Factory, Hangzhou, China) and a pump-suction CO_2_ detector (model PLT300M-CO_2_; Shenzhen Pulitong Electronic Technology Co., Ltd., Shenzhen, China), respectively. For gas measurement, the inlet probe of each instrument was inserted into the package through a sealed sampling port to determine the headspace gas composition. Gas concentrations were expressed as percentages of the total gas volume inside the package.

Respiration intensity was determined according to the method of Long et al. [14]. Briefly, bamboo shoots and a CO_2_ analyzer (model AR8200; HIMA GmbH, Brühl, Germany) were placed together in a sealed container, and changes in CO_2_ concentration in the container headspace during the measurement period were recorded. Respiration intensity was calculated based on the change in CO_2_ concentration, the headspace volume of the container, the measurement time, and the sample weight, using the following formula:

(1) $$\mathrm{Respiratory}\;\mathrm{intensity}\;(\mathrm{mg}\;{\mathrm{CO}}_{2}{\cdot \mathrm{kg}}^{-1}{\cdot h}^{-1})\;=\frac{(C-C_{0})\times V\times \rho }{W\times T\times 1000}$$

where C and C_0_ represent the final and initial CO_2_ volume fractions (mL/m^3^), respectively; W is the weight of bamboo shoots (kg); V is the volume of the desiccator (L); ρ is the density of CO_2_ at room temperature, 1.799 g/L; and T is the incubation time (h).

### 2.3. Color Difference and Cross-Sectional Lignin Staining

The surface color difference of bamboo shoots was determined using an automatic colorimeter (SR-6). A white calibration plate was used as the reference, and the L*, a*, and b* values were measured at the middle section of each bamboo shoot. L* represents lightness (a higher L* value indicates a whiter sample surface), a* represents redness and greenness (positive a* values indicate redness, whereas negative values indicate greenness), and b* represents yellowness and blueness (positive b* values indicate yellowness, whereas negative values indicate blueness). Histochemical staining of lignin was performed using the phloroglucinol–HCl method. Thin transverse sections of bamboo shoots were prepared, followed by the sequential addition of 1% (*w*/*v*) phloroglucinol ethanol solution and concentrated hydrochloric acid. The color development of the Wiesner reaction was then observed.

### 2.4. Decay Rate and Weight Loss Rate

Decay incidence was expressed as the percentage of decayed shoots relative to the total number of assessed shoots. For each biological replicate, eight bamboo shoots were used to determine decay incidence, resulting in 24 shoots per treatment (n = 3). Weight loss was determined using one bamboo shoot per biological replicate, resulting in three shoots per treatment (n = 3). The weight loss rate was calculated based on the change in sample mass before and after storage using the following formula:

(2) $$\mathrm{Weight}\;\mathrm{loss}\;\mathrm{rate}\;(\%)=\frac{W1-W2}{W1}\times 100\%$$

where W1 and W2 represent the initial sample weight and the sample weight after storage, respectively.

### 2.5. Firmness, Lignin, and Cellulose

Firmness was measured using a firmness tester (model LD-GY-4; Hangzhou Lubo Instrument Co., Ltd., Hangzhou, China) and expressed as N. Lignin content was determined using the Klason method [15]. Cellulose content was measured using the acid–alkali detergent method [14]. Briefly, the samples were sequentially boiled with 1.25% (*w*/*v*) H_2_SO_4_ and 1.25% (*w*/*v*) NaOH, followed by filtration, washing, and drying to a constant weight, after which the cellulose content was calculated.

### 2.6. MDA, SOD, CAT, and APX

MDA content was determined using the thiobarbituric acid method [16]. Briefly, 1.0 g of bamboo shoot sample was weighed and mixed with 5.0 mL of a 100 g/L trichloroacetic acid (TCA) solution. The mixture was ground and homogenized and then centrifuged at 10,000× *g* for 20 min at 4 °C. The supernatant was collected and stored at low temperature for subsequent analysis. Then, 2.0 mL of the supernatant was mixed with 2.0 mL of 0.67% (*w*/*v*) thiobarbituric acid (TBA). For the blank control tube, 2.0 mL of a 100 g/L TCA solution was added instead of the extract. After mixing, the reaction mixture was boiled in a water bath for 20 min, cooled, and centrifuged again. The absorbance of the supernatant was measured at 450, 532, and 600 nm, respectively.

SOD and CAT activities were determined according to the method of Wang et al. [5]. The absorbance for SOD activity was measured at 560 nm, and one unit of enzyme activity was defined as the amount of enzyme required to inhibit the photoreduction of nitroblue tetrazolium by 50%. CAT activity was measured at 240 nm, and one unit of activity was defined as a decrease in absorbance of 0.01 per minute per gram of fresh bamboo shoot weight. APX activity was determined according to the method of Corpas et al. [17]. Briefly, 2.6 mL of reaction buffer and 0.1 mL of enzyme extract were added sequentially, followed by the addition of 0.3 mL of 2 mM H_2_O_2_ solution to initiate the enzymatic reaction. One unit of APX activity was defined as a decrease in absorbance of 0.01 per minute per gram of fresh bamboo shoot weight. All results were expressed as U/g.

### 2.7. PAL, POD, and PPO

PAL activity was determined according to the method of Kahramanoğlu et al. [18]. Briefly, 3.0 mL of 50 mM borate buffer and 0.5 mL of 20 mM phenylalanine solution were added, and the mixture was incubated in a water bath at 37 °C for 60 min. Distilled water was used as the reference blank for zero adjustment, and the absorbance of the sample was measured at 290 nm. The assay was performed in triplicate. One unit of PAL activity was defined as an increase in absorbance of 0.01 per hour per gram of fresh sample weight.

POD activity was determined according to the method of Long et al. [14]. Briefly, 3.0 mL of 0.25 mM guaiacol solution and 0.5 mL of enzyme extract were added, followed by the addition of 200 µL of 0.01 mol/L H_2_O_2_ solution. The mixture was rapidly mixed to initiate the reaction. Distilled water was used as the reference, and the absorbance of the sample was measured at 470 nm. The assay was performed in triplicate. One unit of POD activity was defined as an increase in absorbance of 1 per minute per gram of fresh sample weight.

PPO activity was determined according to the method of Yang et al. [19], with slight modifications. Briefly, 4.0 mL of 100 mM acetate–sodium acetate buffer (pH 5.5) and 1.0 mL of 50 mM catechol solution were added, followed by the addition of 100 µL of enzyme extract, and timing was started immediately. Distilled water was used as the reference, and the absorbance of the sample was recorded at 420 nm. The assay was performed in triplicate. One unit of PPO activity was defined as an increase in absorbance of 1 per minute per gram of fresh bamboo shoot weight.

### 2.8. Untargeted Metabolomics and Microbial Diversity

Untargeted metabolomic analysis and microbial diversity sequencing were performed by Wuhan Kangce Technology Co., Ltd. For these analyses, three sample groups were included: CK1, fresh arrow bamboo shoot samples at day 0; CK2, untreated control samples after 14 d of storage at 4 °C; and EM2, EFW+MA-treated samples after 14 d of storage at 4 °C. Hereafter, “EFW+MA” refers to the treatment condition applied throughout the storage experiment, whereas “EM2” refers specifically to the day-14 sample group from the EFW+MA treatment used for metabolomic and microbial analyses.

The day-0 samples represented the common initial metabolic state of the arrow bamboo shoots before storage. The day-14 sampling point was selected because clear differences in physiological and quality-related parameters between the CK and EFW+MA groups had emerged, while the shoots still retained acceptable quality. Three independent biological replicates were prepared for each group, resulting in a total of nine samples. After sampling, all samples were immediately frozen in liquid nitrogen and stored at −80 °C until further analysis.

Untargeted metabolomics was performed using liquid chromatography–tandem mass spectrometry (LC-MS/MS) and gas chromatography–mass spectrometry (GC-MS). Frozen bamboo shoot samples were ground into a fine powder under liquid nitrogen. For LC-MS/MS analysis, 100 mg of powdered sample was extracted with 1.0 mL of prechilled 70% methanol *v*/*v*. The mixture was vortexed, sonicated in an ice-water bath for 30 min, and centrifuged at 12,000× *g* for 15 min at 4 °C. The supernatant was collected and filtered through a 0.22 μm membrane filter before LC-MS/MS analysis. Quality-control samples were prepared by pooling equal aliquots of extracts from all samples and were analyzed periodically to monitor analytical stability. Subsequently, peak extraction, peak alignment, normalization, and metabolite annotation were performed. For GC-MS analysis, an aliquot of the extract was dried under vacuum and derivatized sequentially with methoxyamine hydrochloride in pyridine and N-methyl-N-trifluoroacetamide before analysis. Multivariate statistical methods, including principal component analysis (PCA) and orthogonal partial least squares-discriminant analysis (OPLS-DA), were used to analyze differences in metabolic profiles among different samples. Differential metabolites were screened according to variable importance in projection (VIP) scores, fold changes, and significance levels, followed by metabolic pathway enrichment analysis.

Microbial diversity analysis was performed using high-throughput sequencing. After total deoxyribonucleic acid (DNA) extraction, specific regions of the bacterial 16S rRNA gene were amplified by polymerase chain reaction (PCR). The sequencing data were subjected to quality control, sequence assembly, denoising, and taxonomic annotation, followed by analysis of microbial community composition, alpha diversity (α-diversity), beta diversity (β-diversity), and differences in species abundance at different taxonomic levels among the samples.

### 2.9. Untargeted Metabolomics and Microbial Diversity Data Analysis

Metabolomic data were subjected to peak extraction, peak alignment, normalization, and metabolite annotation before statistical analysis. PCA was used to visualize overall differences in metabolic profiles among groups, whereas OPLS-DA was used to distinguish between the CK2 and EM2 groups. The robustness of the OPLS-DA model was evaluated using a 200-permutation test. Differential metabolites were screened using VIP > 1 and |log_2_FC| > 1. The normalized abundances of differential metabolites were Z-score standardized for hierarchical clustering analysis. KEGG pathway enrichment analysis was performed to characterize the metabolic pathways associated with the differential metabolites. Pearson’s correlation coefficients were calculated to evaluate associations among differential metabolites. Three independent biological replicates were analyzed for each group (n = 3).

For microbial-community analysis, the 16S rRNA gene-sequencing data were subjected to quality control, sequence assembly, denoising, operational taxonomic unit (OTU) identification, and taxonomic annotation. OTU richness, taxonomic composition at the phylum and family levels, and the Shannon, Simpson, and InvSimpson alpha diversity indices were calculated. Pearson’s correlation coefficients were used to evaluate similarities among the microbial-community profiles of the samples. Three independent biological replicates were analyzed for each group (n = 3).

### 2.10. Data Analysis

Physiological and quality-related data were analyzed by two-way analysis of variance (ANOVA), with treatment and storage time as fixed factors. When a significant treatment × storage time interaction was detected, pairwise comparisons between CK and EFW+MA at each storage time were performed using Tukey–Kramer-adjusted comparisons. Differences were considered significant at *p* < 0.05. All data represent three independent biological replicates and are presented as mean ± standard deviation (SD). Figures were prepared using Origin 2026.

## 3. Results and Discussion

### 3.1. Changes in Appearance and Gas Concentration Inside the Packaging Bags During Storage

**Figure 1:**
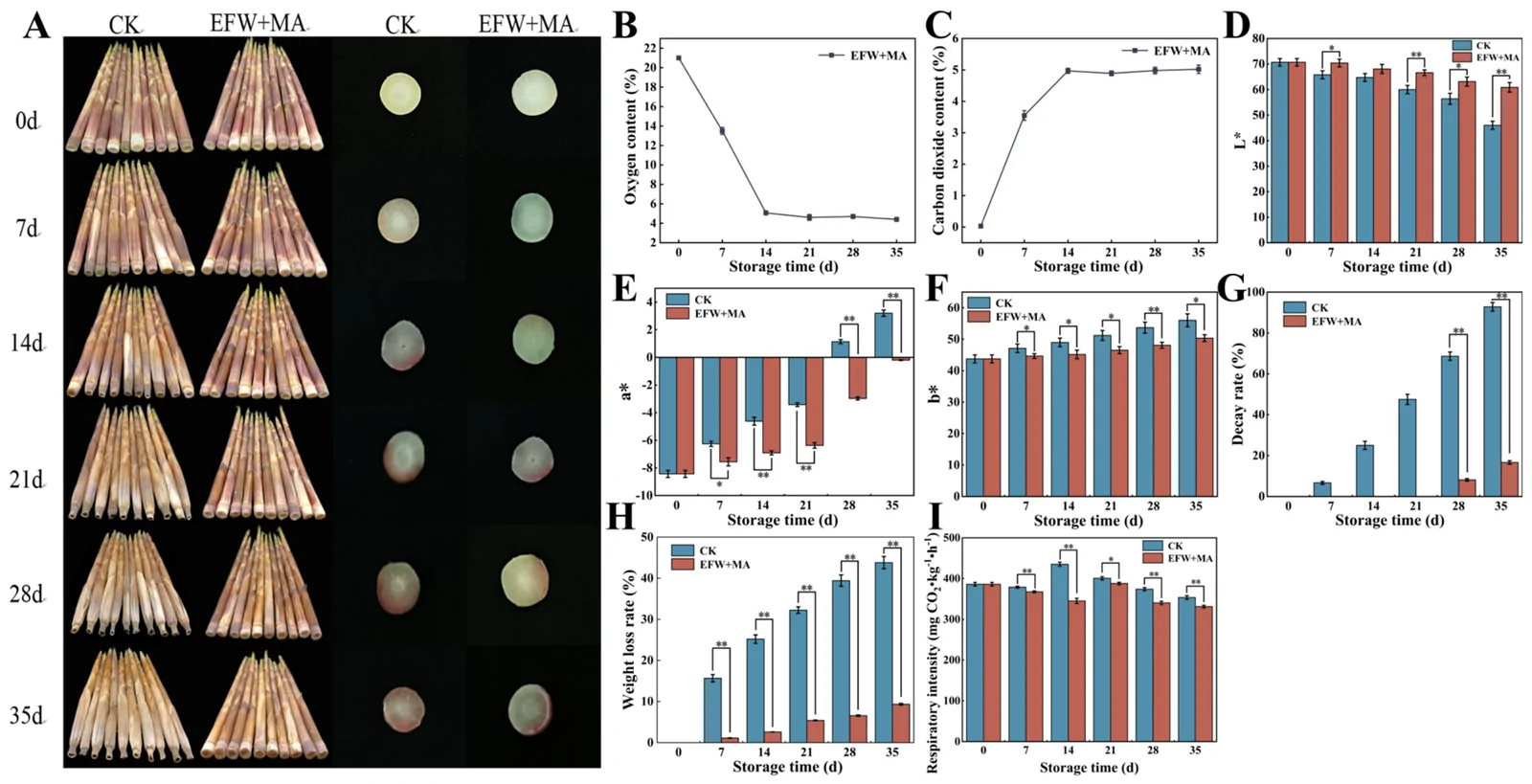
Effects of EFW+MA treatment on the visual quality and physical characteristics of arrow bamboo shoots during storage: (**A**) appearance and lignin histochemical staining; (**B**) O_2_ content inside the packaging; (**C**) CO_2_ content inside the packaging; (**D**) L* value; (**E**) a* value; (**F**) b* value; (**G**) decay rate; (**H**) weight loss rate; (**I**) respiration intensity. Data are presented as mean ± SD. * *p* < 0.05, ** *p* < 0.01.

As shown in Figure 1A, arrow bamboo shoots exhibited obvious visual deterioration and lignification during refrigerated storage. In the CK group, water loss and wilting, yellowing and browning, and basal shrinkage gradually occurred with prolonged storage, with particularly severe deterioration observed at 28–35 d. The staining intensity of the cross sections gradually increased, indicating increased lignin deposition during storage. In contrast, the EFW+MA group better maintained shoot integrity and a fresh, tender appearance throughout the storage period, and the degree of browning and staining in the cross-sections was markedly lower than that in the CK group. These results demonstrate that EFW+MA delayed postharvest lignification and preserved the visual quality of arrow bamboo shoots.

The gas composition inside the packaging showed that (Figure 1B,C) the O_2_ concentration in the EFW+MA group rapidly decreased from approximately 21% to below 5%, while the CO_2_ concentration increased to approximately 5% and remained stable, indicating the formation of a spontaneous modified atmosphere characterized by low O_2_ and high CO_2_. Such a microenvironment is generally considered favorable for limiting respiratory substrate consumption and delaying senescence in fresh produce [5,13].

The color difference results further demonstrated that EFW+MA significantly inhibited color deterioration. Although L* decreased and a* and b* increased in both groups during storage, the treated shoots retained higher L* values and showed smaller changes in a* and b* than the CK group (Figure 1D–F), indicating better maintenance of visual color. The greater yellowing and browning in CK samples may be related to chlorophyll degradation and oxidative browning [20]. In addition, EFW+MA significantly reduced decay rate, weight loss rate, and respiration intensity (Figure 1G–I). At 35 d, decay incidence was 92.78 ± 2.10% in the CK group but only 16.67 ± 0.84% in the treated group. Together, these results suggest that EFW washing and MA packaging may provide complementary preservation effects, with EFW potentially reducing initial microbial contamination and MA helping maintain a favorable storage atmosphere.

### 3.2. Changes in MDA Content and Antioxidant-Related Enzyme Activities During Storage

**Figure 2:**
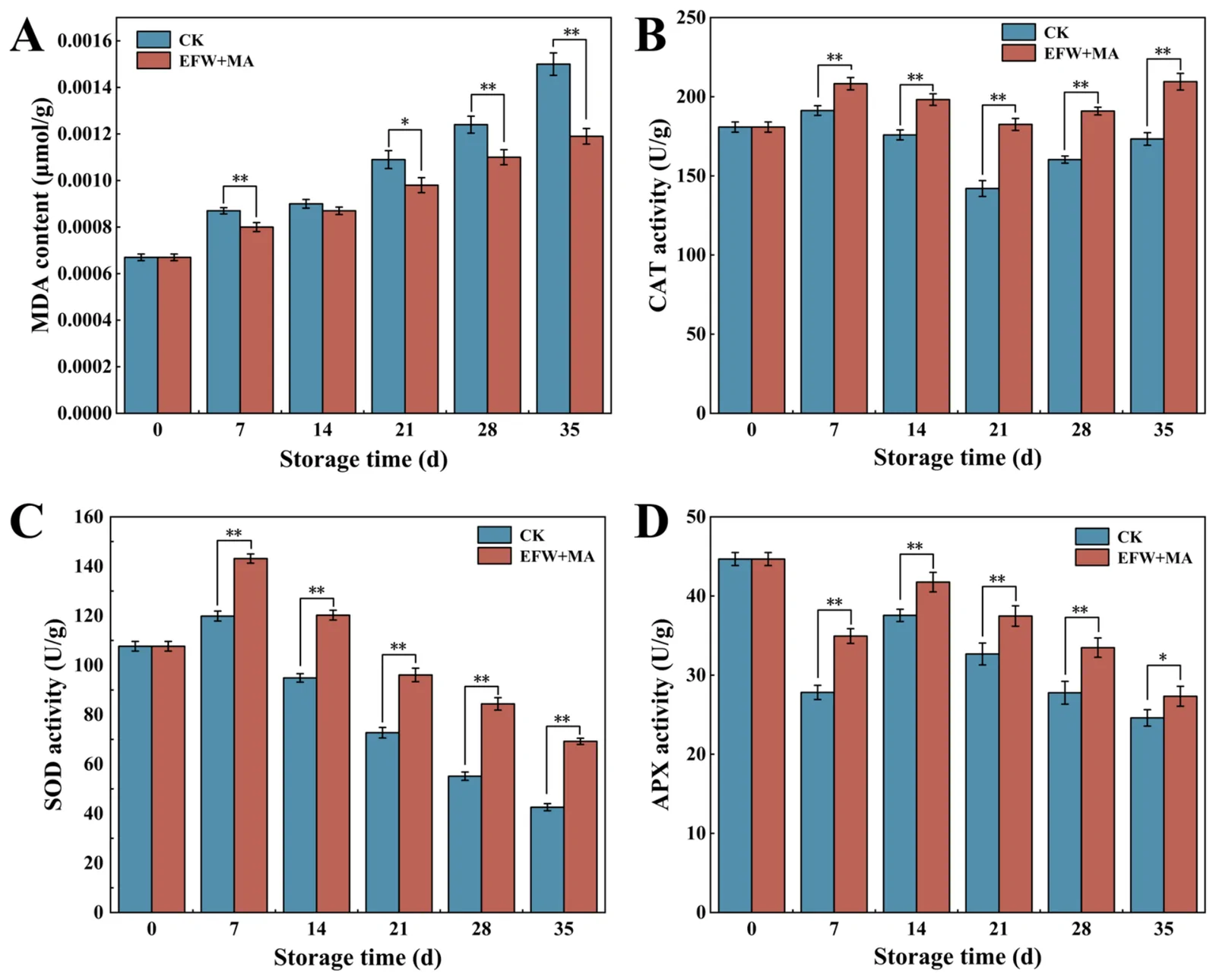
Effects of EFW+MA treatment on membrane lipid peroxidation and antioxidant enzyme activities in arrow bamboo shoots during storage: (**A**) MDA content; (**B**) CAT activity; (**C**) SOD activity; (**D**) APX activity. Data are presented as mean ± SD. * *p* < 0.05, ** *p* < 0.01.

Membrane lipid peroxidation is an important manifestation of postharvest senescence and membrane damage, and MDA, as a major end product, reflects the extent of oxidative injury to cell membranes [21]. The lower MDA content in EFW+MA-treated shoots, especially during the later storage period (Figure 2A), was consistent with alleviated oxidative membrane damage. Consistent with the present results, EFW treatment alleviated chilling injury in Satsuma mandarin fruit during cold storage, which was accompanied by lower MDA content and reduced membrane lipid peroxidation [22].

Meanwhile, CAT, SOD, and APX activities were higher in the EFW+MA-treated group at most time points (Figure 2B–D). SOD converts superoxide anions into H_2_O_2_, whereas CAT and APX subsequently decompose H_2_O_2_, thereby limiting the potential formation of more damaging reactive species [23]. The maintenance of APX activity may also be associated with the ascorbate–glutathione cycle [24]. Therefore, the coordinated changes in SOD, CAT, and APX activities may have contributed to the lower MDA accumulation observed in treated shoots. This interpretation is consistent with the findings of He et al. [25], who reported increased SOD and CAT activities in fresh goji berries treated with electrolyzed water. Overall, EFW+MA treatment delayed postharvest quality deterioration of arrow bamboo shoots by reducing MDA accumulation and maintaining higher antioxidant enzyme activities.

### 3.3. Changes in Texture- and Lignification-Related Physiological Indicators During Storage

**Figure 3:**
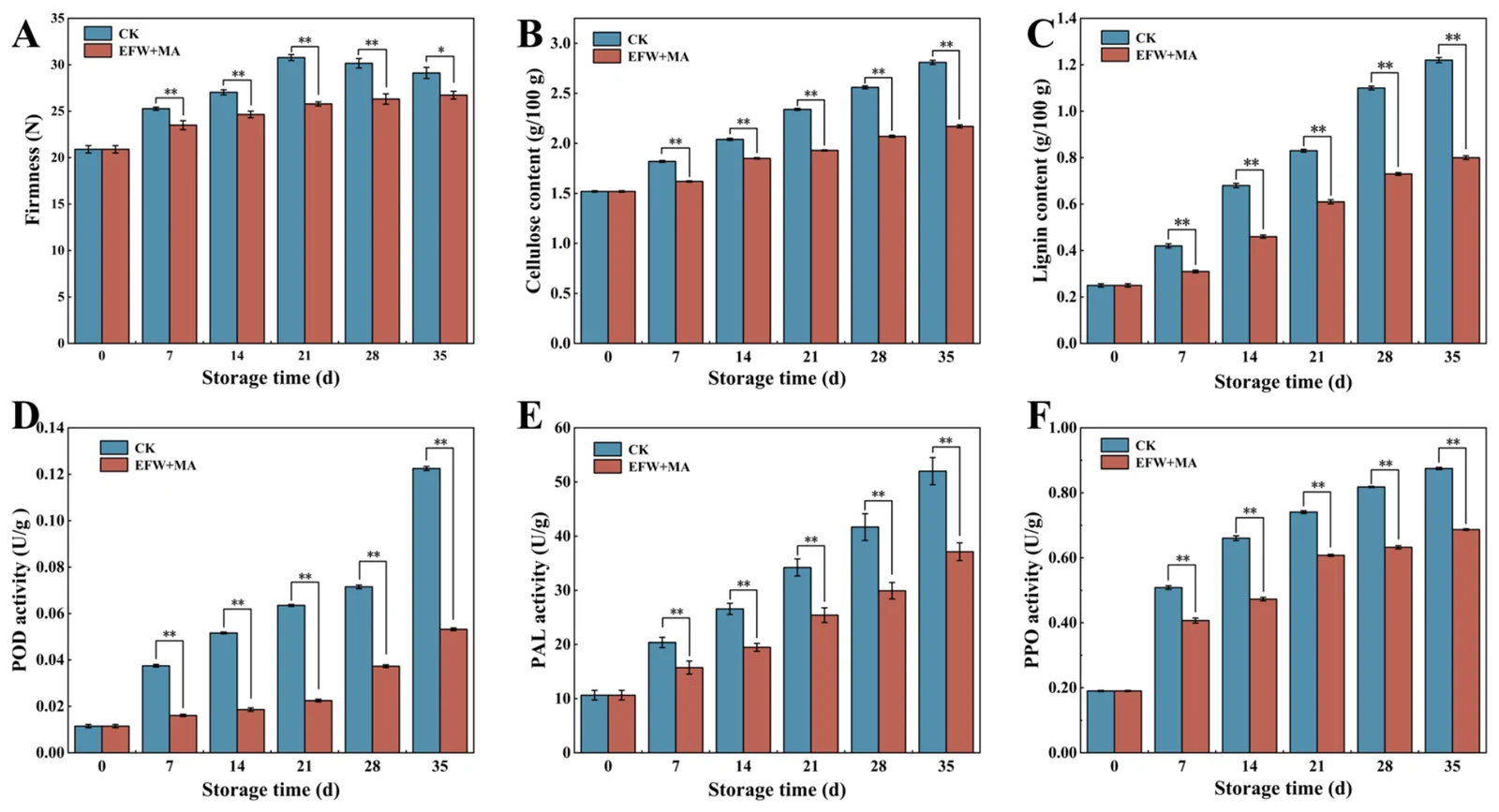
Effects of EFW+MA treatment on texture- and lignification-related physiological indicators in arrow bamboo shoots during storage: (**A**) shoot firmness; (**B**) cellulose content; (**C**) lignin content; (**D**) POD activity; (**E**) PAL activity; (**F**) PPO activity. Data are presented as mean ± SD. * *p* < 0.05, ** *p* < 0.01.

As shown in Figure 3, postharvest quality deterioration of arrow bamboo shoots was characterized primarily by tissue hardening and lignification rather than the softening commonly observed in many vegetables. The lower firmness and reduced accumulation of cellulose and lignin in EFW+MA-treated shoots (Figure 3A–C) suggest that the treatment helped preserve edible texture by limiting cell wall reinforcement during storage. This result is consistent with the finding of Xu et al. [26] that 1-methylcyclopropene (1-MCP) combined with sulfur dioxide (SO_2_) delayed textural deterioration in bamboo shoots. Moreover, EFW+MA provides a residue-free preservation approach with potential practical value, as related combined treatments have also been reported to maintain postharvest quality in strawberries and jujubes [27,28].

The differences in PAL, POD, and PPO activities between the two groups (Figure 3D–F) were consistent with the observed differences in lignin accumulation and tissue hardening. PAL is associated with phenylpropanoid metabolism, whereas POD and PPO are related to phenolic oxidation and lignin monomer polymerization [19]. In particular, POD is considered an important enzyme involved in the final polymerization of lignin monomers in bamboo shoots [2]. Similar associations between lower POD activity and delayed lignification have been reported in melatonin-treated bamboo shoots [5], and acidic electrolyzed water has also been associated with changes in enzymatic reactions during postharvest storage [29]. Overall, EFW+MA treatment significantly delayed postharvest hardening and lignification of arrow bamboo shoots by reducing cellulose and lignin accumulation and suppressing POD, PAL, and PPO activities.

### 3.4. Metabolomic Analysis of Arrow Bamboo Shoots

#### 3.4.1. Integrated Metabolomic Analysis of Postharvest Arrow Bamboo Shoots

**Figure 4:**
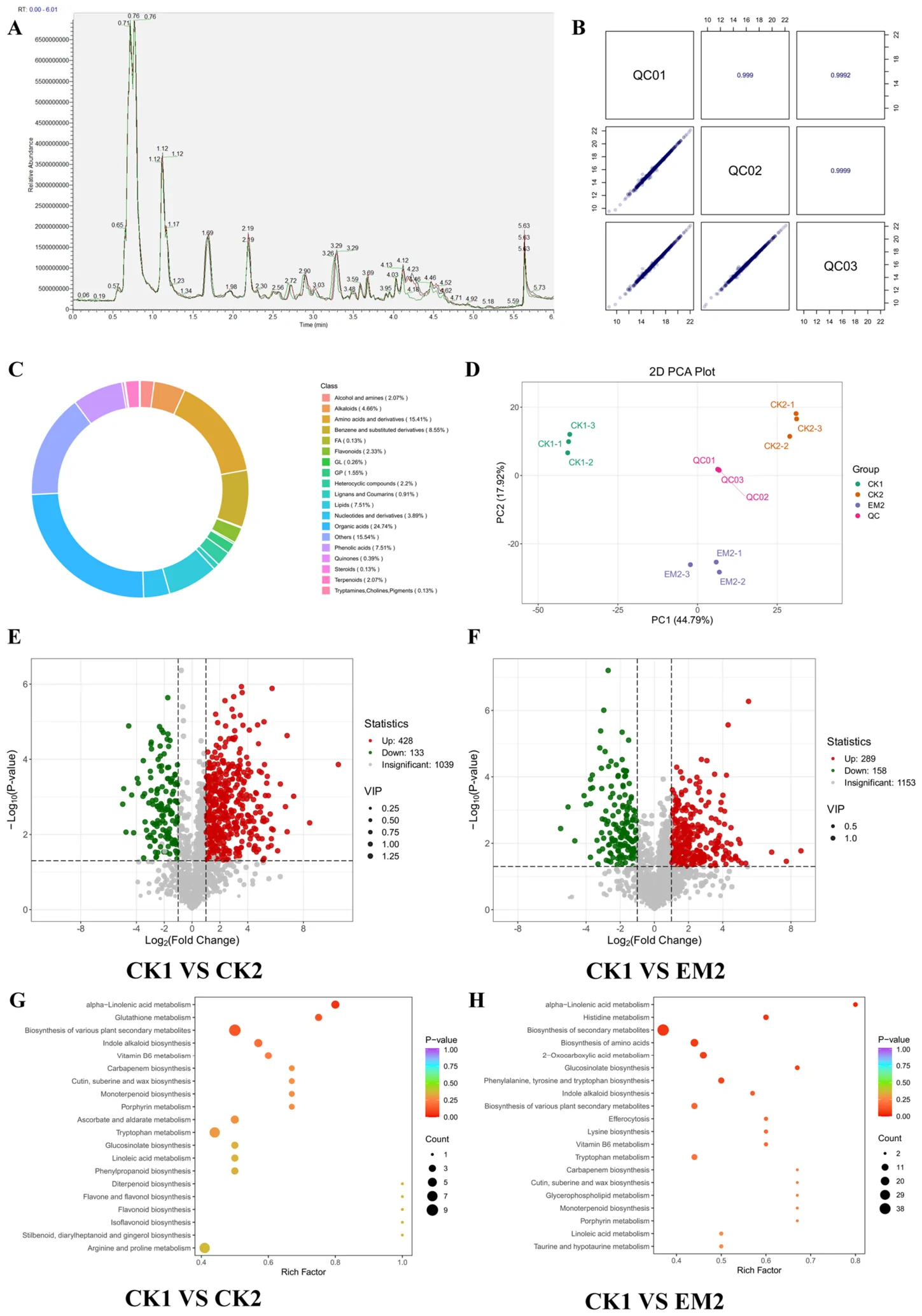
Metabolomic analysis of arrow bamboo shoots: (**A**) total ion chromatogram (TIC) of samples; (**B**) correlation analysis of QC samples; (**C**) donut chart showing the category distribution of metabolites detected across the full spectrum; (**D**) PCA score plot of all samples; (**E**) volcano plot of differential metabolites in CK1 vs. CK2; (**F**) volcano plot of differential metabolites in CK1 vs. EM2; (**G**) KEGG enrichment bubble plot of differential metabolites in CK1 vs. CK2; (**H**) KEGG enrichment bubble plot of differential metabolites in CK1 vs. EM2.

To characterize metabolite-profile changes associated with EFW+MA treatment and postharvest quality in arrow bamboo shoots, LC-MS/MS-based untargeted metabolomics was performed. The total ion chromatogram (TIC) overlay and correlation analysis of quality-control (QC) samples showed good peak overlap and correlation coefficients close to 1 (Figure 4A,B), indicating stable analysis and reliable data. A total of 1600 metabolites were identified, including 904 in positive-ion mode and 696 in negative-ion mode. These metabolites mainly included organic acids (24.74%), amino acids and their derivatives (15.41%), benzene and substituted derivatives (8.55%), lipids (7.51%), and phenolic acids (7.51%) (Figure 4C).

Principal component analysis (PCA) can be used to evaluate metabolic differences among groups and reproducibility within groups [30]. PCA showed clear separation among CK1, CK2, and EM2, whereas biological replicates clustered well within each group (Figure 4D), indicating that both storage and EFW+MA markedly altered the metabolic profile of arrow bamboo shoots. In CK1 vs. CK2, 428 metabolites were upregulated, and 133 were downregulated; in CK1 vs. EM2, 289 metabolites were upregulated, and 158 were downregulated (Figure 4E,F). The lower number of upregulated metabolites in EM2 indicated that EFW+MA attenuated storage-associated metabolic changes.

Kyoto Encyclopedia of Genes and Genomes (KEGG) enrichment analysis showed that differential metabolites in CK2 were mainly enriched in alpha-linolenic acid metabolism, glutathione metabolism, biosynthesis of secondary metabolites, and indole alkaloid biosynthesis, whereas those in EM2 were enriched in alpha-linolenic acid metabolism, phenylalanine biosynthesis, tyrosine and tryptophan biosynthesis, 2-oxocarboxylic acid metabolism, and biosynthesis of secondary metabolites (Figure 4G,H). Phenylalanine is an important precursor of phenylpropanoid metabolism and lignin-related phenolic compounds. Therefore, the changes in phenylalanine-related and secondary metabolite pathways were consistent with the increased PAL and POD activities, lignin content, cellulose content, and firmness in CK2. In contrast, the lower PAL and POD activities and reduced lignin accumulation in EM2 were associated with differences in phenylalanine-related and secondary metabolite profiles. These observations were consistent with less pronounced lignification-associated physiological changes in the treated samples; however, they do not demonstrate that EFW+MA directly altered lignification-related metabolic flux. The observed pathway changes were consistent with reduced precursor formation and oxidative polymerization. In addition, alpha-linolenic acid metabolism is associated with membrane lipid turnover. Its enrichment in stored samples, together with the higher MDA content in CK2, was consistent with enhanced lipid peroxidation during storage. The lower MDA content and higher SOD, CAT, and APX activities in EM2 indicated that EFW+MA alleviated oxidative membrane damage. Moreover, the higher PPO and POD activities and lower L* values in CK2 were consistent with enhanced phenolic oxidation and browning. Thus, EFW+MA delayed browning, concomitant with lower PPO and POD activities and reduced phenolic-oxidation-associated metabolic changes. These results indicate treatment-associated metabolic differences related to lignification, browning, and lipid peroxidation. However, untargeted metabolomics and pathway enrichment do not directly demonstrate metabolic flux, gene regulation, enzyme regulation, or causal relationships.

#### 3.4.2. Cluster Analysis of Differential Metabolites in Arrow Bamboo Shoots During Storage

**Figure 5:**
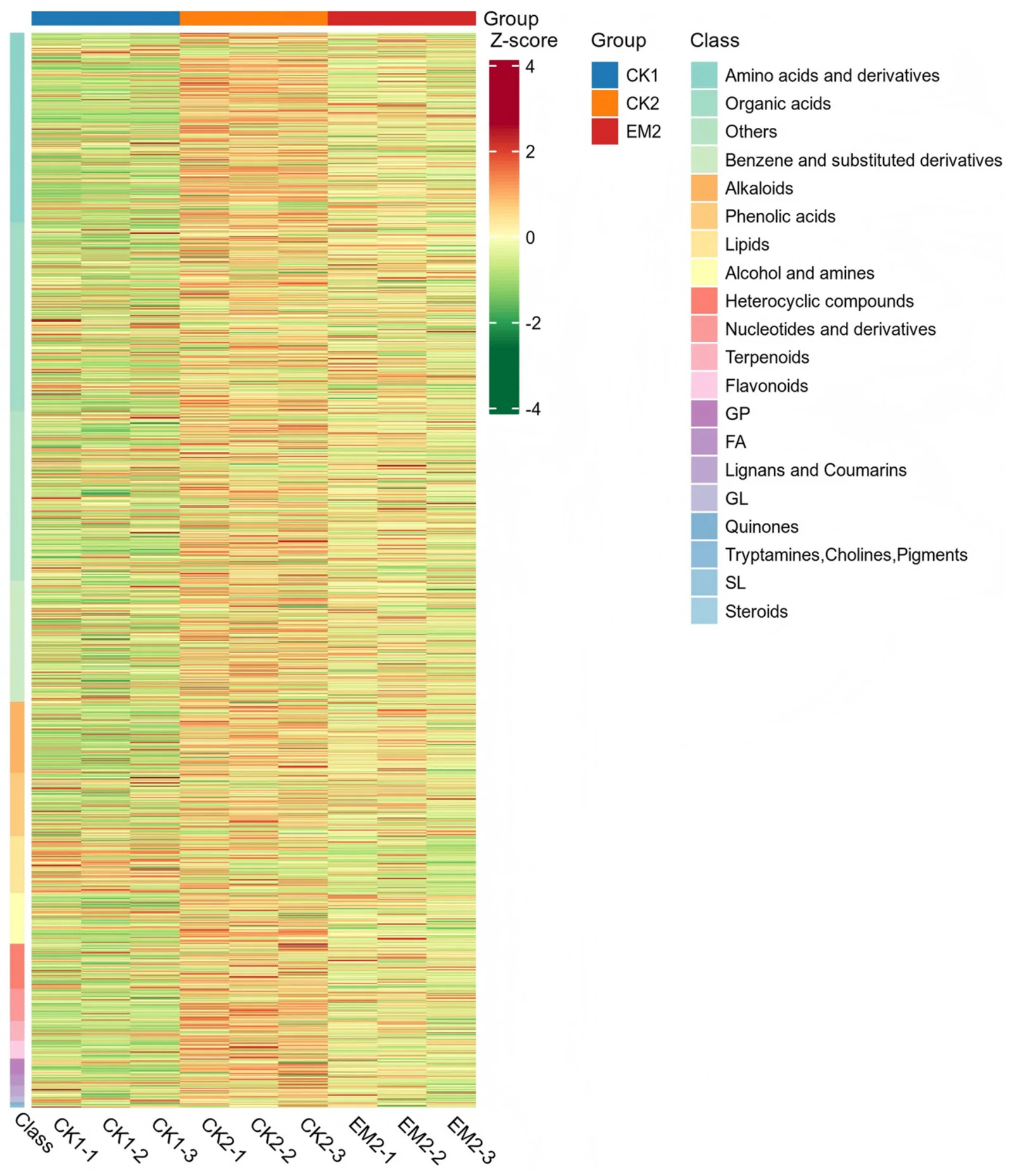
Z-score-standardized expression clustering heatmap of differential metabolites in arrow bamboo shoots.

The Z-score-standardized metabolite heatmap (Figure 5) shows that the replicate samples within the CK1, CK2, and EM2 groups exhibited generally consistent chromatographic patterns, indicating good reproducibility and stability of the metabolomic data. In contrast, clear metabolic differentiation was observed among the different treatment groups. CK1 samples were mainly characterized by green and light-yellow colors, suggesting that most metabolites in fresh arrow bamboo shoots were present at low or moderate abundance levels. After storage, a large number of metabolites in CK2 shifted to yellow and red, particularly among amino acids and their derivatives, organic acids, phenolic acids, lipids, and alkaloids. This metabolite pattern was consistent with storage-associated changes in respiratory substrate consumption, membrane lipid remodeling, phenolic oxidation, and secondary metabolism.

Similarly, an untargeted LC-MS metabolomics study reported that fresh-cut tissues can undergo a transition from enzymatic browning to lignification-related metabolism during modified-atmosphere storage, involving phenolic metabolism, cell wall reinforcement, and reprogramming of defense responses [31]. Compared with CK2, some high-abundance metabolite signals were weakened in EM2, and overall metabolic fluctuations tended to be alleviated, indicating that EFW+MA reduced excessive metabolic activation during the later stage of storage.

Notably, postharvest lignification in bamboo shoot products is generally closely associated with ROS signaling, the phenylpropanoid pathway, and the polymerization of lignin monomers. Respiratory burst oxidase homolog (RBOH)-mediated ROS accumulation can further drive lignin deposition [32]. Therefore, the delayed quality deterioration observed in EFW+MA-treated shoots was accompanied by lower MDA accumulation, higher antioxidant-enzyme activities, and less pronounced changes in primary and secondary metabolite profiles associated with browning and lignification.

#### 3.4.3. Changes in the Contents of Volatile Flavor Compounds in Arrow Bamboo Shoots During Storage

**Figure 6:**
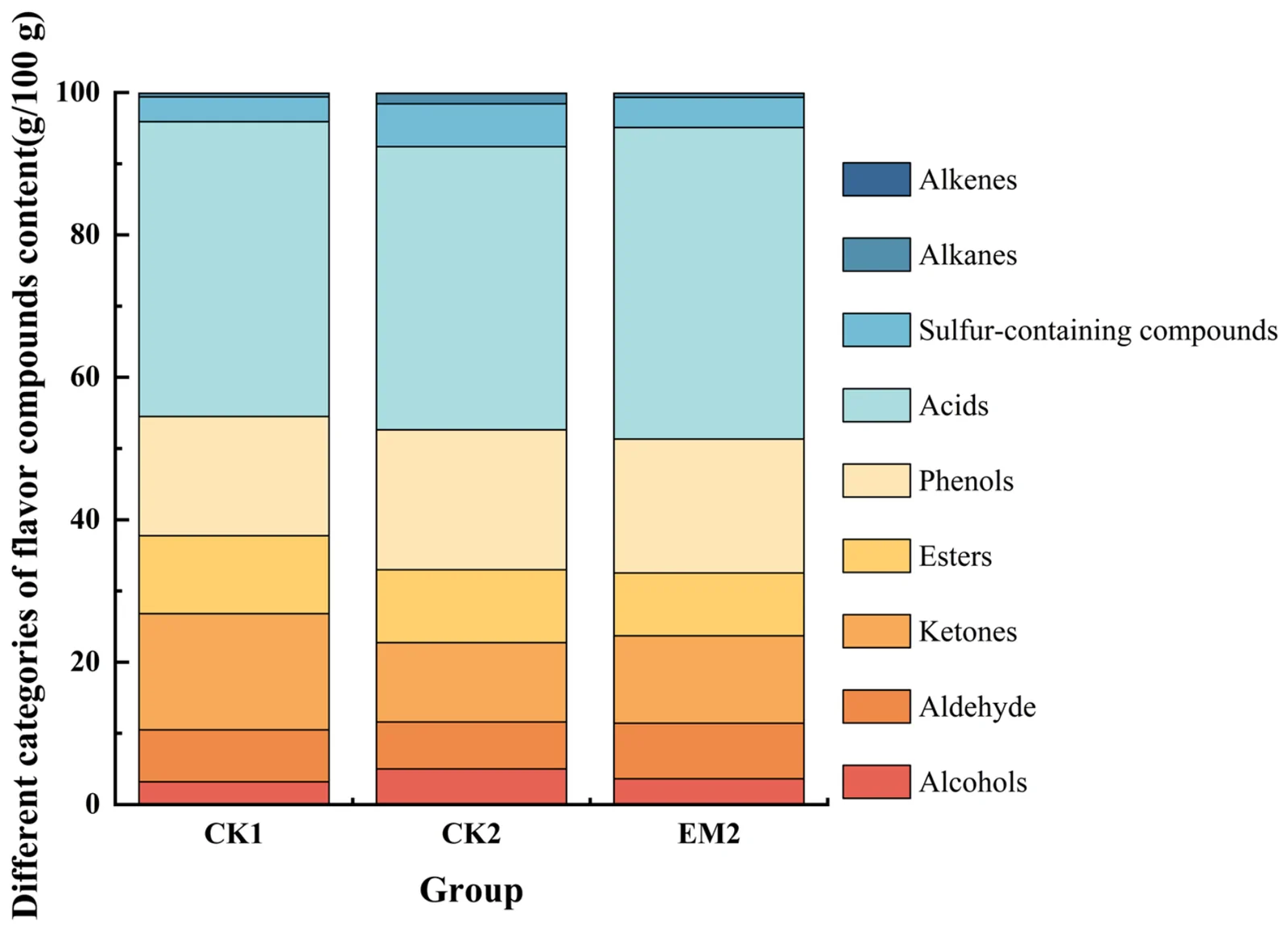
Relative contents of different volatile flavor compound categories in arrow bamboo shoots across the CK1, CK2 and EM2 groups.

The volatile composition of bamboo shoots is complex and can vary with cultivar, storage conditions, and processing method [33]. Alcohols, aldehydes, ketones, esters, phenols, acids, sulfur-containing compounds, alkanes, and alkenes were detected in arrow bamboo shoots (Figure 6). These compounds may originate from amino acid metabolism, carbohydrate metabolism, fatty acid oxidation, phenolic metabolism, and microbial activity [34]. In fresh samples (CK1), acids, phenols, and ketones accounted for 41.38%, 16.74%, and 16.30%, respectively, indicating that these were the predominant volatile categories.

After 14 days of storage, the contents of acids, phenols, aldehydes, and ketones increased in CK2. Aldehydes and ketones are common products of fatty acid degradation and lipid oxidation [35]; thus, their increase was consistent with the higher MDA content in CK2 and was consistent with a contribution of membrane lipid peroxidation to changes in the volatile profile. Aldehydes are commonly associated with green, grassy, fatty, or rancid notes, whereas some ketones may contribute fatty, mushroom-like, or fermented notes. In addition, the increase in acids and sulfur-containing compounds may be related to microbial metabolism and tissue degradation, as these compounds are often associated with sour, pungent, sulfurous, or off-odor characteristics [36]. This interpretation was consistent with the higher decay rate and microbial richness in CK2. In contrast, EM2 showed lower contents of aldehydes, esters, phenols, and sulfur-containing compounds than CK2, which was consistent with lower MDA accumulation and reduced microbial richness. Therefore, EFW+MA maintained a relatively stable volatile profile, concomitant with lower MDA accumulation and reduced microbial richness. However, as sensory evaluation, GC-olfactometry, and odor activity value analyses were not performed, the present results cannot identify an individual compound as directly responsible for flavor alteration.

#### 3.4.4. Differential Metabolomic Analysis of Arrow Bamboo Shoots in the CK2 and EM2 Groups

**Figure 7:**
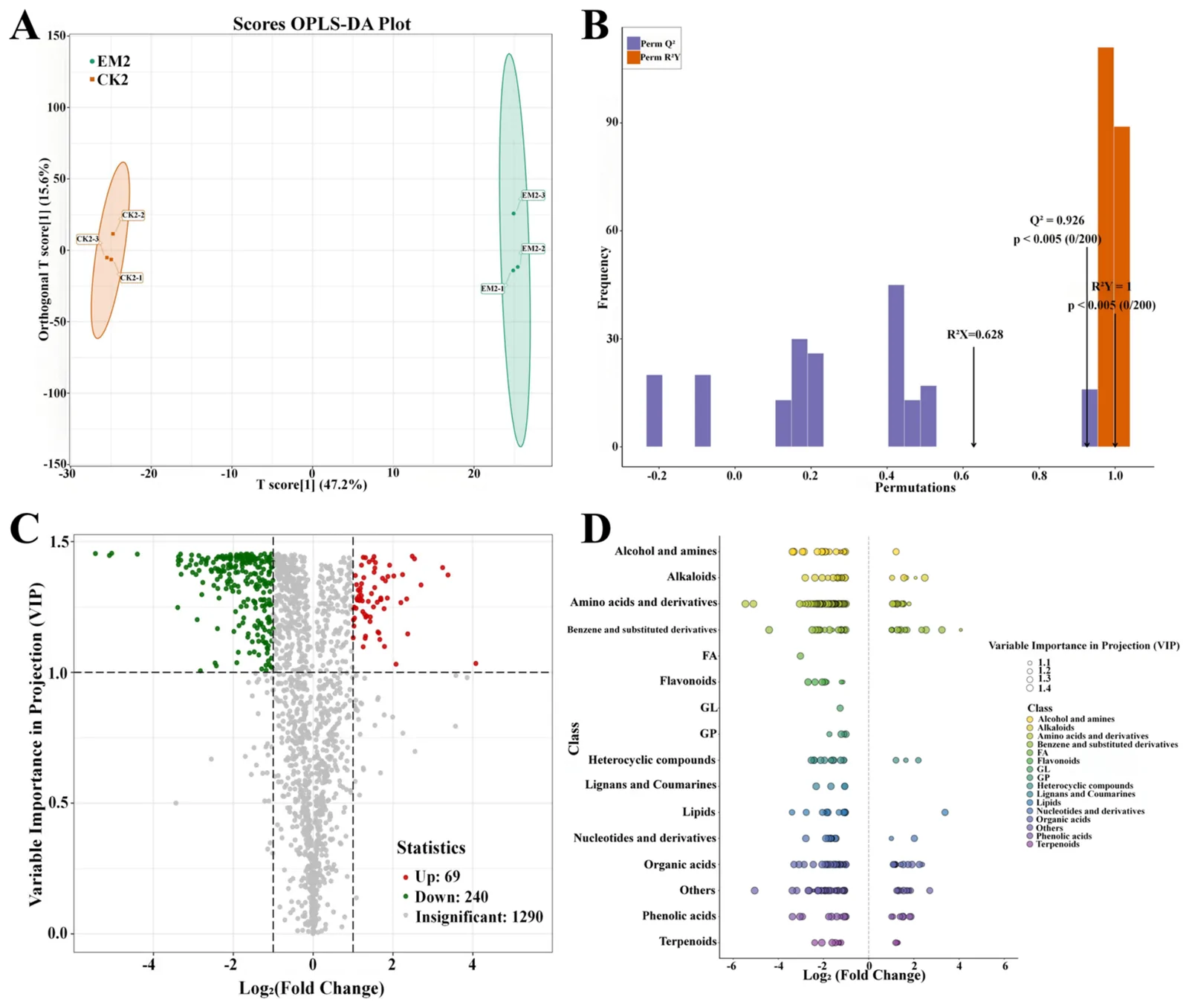
Metabolic profile differences between CK2 and EM2 and the screening and classification of differential metabolites: (**A**) OPLS-DA score plot; (**B**) validation of model reliability by a 200-times permutation test; (**C**) VIP volcano plot of differential metabolites (screening criteria: VIP > 1 and |Log_2_FC| > 1); (**D**) distribution of VIP values and Log_2_FC of differential metabolites in different categories.

CK2 and EM2 were clearly separated in the OPLS-DA model, with good clustering of biological replicates within each group, indicating that EFW+MA treatment significantly altered the metabolic profile of arrow bamboo shoots at the later stage of storage (Figure 7A). The permutation test showed that both R^2^Y and Q^2^ values of the model were high, and the Q^2^ intercept after permutation was lower than that of the original model, indicating that the model had good explanatory power and predictive stability, with no obvious overfitting (Figure 7B).

Based on VIP > 1 and fold-change screening, a total of 309 differential metabolites were identified, including 69 metabolites with higher abundance and 240 metabolites with lower abundance in EM2 than in CK2 (Figure 7C). This distribution suggested that EFW+MA treatment was associated with less extensive accumulation of many storage-responsive metabolites. These differential metabolites were mainly distributed among categories such as organic acids, amino acids and their derivatives, benzene and substituted derivatives, phenolic acids, lipids, alkaloids, and nucleotide derivatives (Figure 7D). These compounds are closely associated with respiratory metabolism, membrane lipid oxidation, phenolic metabolism, and cell wall lignification.

Previous untargeted LC-MS metabolomics studies have shown that postharvest plant tissues undergo a metabolic shift from enzymatic browning to lignification-related metabolism during modified-atmosphere storage, accompanied by the reprogramming of phenolic and cell wall-associated metabolic pathways [30]. Therefore, the delayed quality deterioration in EFW+MA-treated arrow bamboo shoots was accompanied by differences in amino acids, organic acids, lipids, and phenolic acids associated with browning and lignification.

#### 3.4.5. Correlation-Network Analysis of Differential Metabolites in Arrow Bamboo Shoots from the CK2 and EM2 Groups

**Figure 8:**
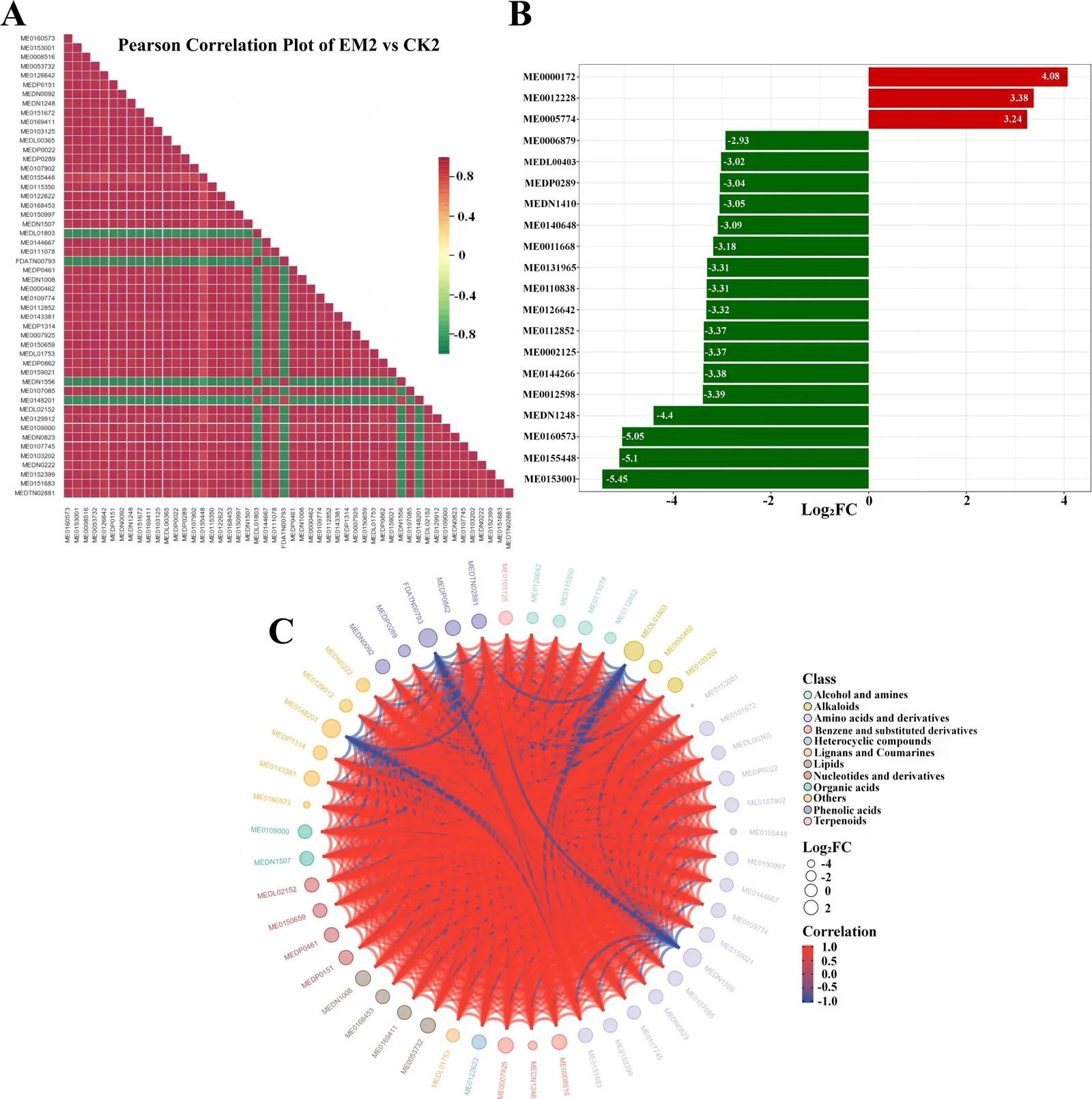
Correlation analysis and metabolite correlation network of differential metabolites in CK2 and EM2: (**A**) Pearson correlation heatmap of core differential metabolites in the EM2 vs. CK2 comparison; (**B**) bar chart of Log_2_FC values for the top 20 most significantly differential metabolites (red indicates upregulated metabolites and green indicates downregulated metabolites); (**C**) Circos co-expression network of differential metabolites (red indicates positive correlation, and blue indicates negative correlation).

Correlation analysis of differential metabolites further characterized the coordinated variation patterns among metabolites in arrow bamboo shoots during storage. The Pearson correlation heatmap showed that most differential metabolites in the EM2 versus CK2 comparison were significantly positively correlated with each other, while only a small number formed negatively correlated modules, indicating that metabolite changes at the late stage of storage exhibited strong coordinated response patterns (Figure 8A). The Log_2_FC ranking results showed that only a few metabolites were significantly upregulated in EM2, whereas most key differential metabolites were markedly downregulated, with the largest decrease showing a Log_2_FC below −5. These results showed that many differential metabolites had lower relative abundances in EM2 than in CK2, indicating that EFW+MA treatment was associated with less pronounced storage-related changes in the metabolite profile (Figure 8B).

The correlation network diagram showed complex interactions among different classes of metabolites. Amino acids and their derivatives, organic acids, phenolic acids, lipids, alkaloids, and nucleotide derivatives constituted the major network nodes, and most connections were positively correlated, indicating that these metabolic pathways may jointly participate in the postharvest senescence, browning, and lignification processes of arrow bamboo shoots (Figure 8C). Some negative correlations indicate that EFW+MA was associated with altered coordination among carbon–nitrogen metabolism, phenolic metabolism, and membrane lipid metabolism. Overall, EFW+MA treatment was associated with coordinated changes across multiple metabolite classes and better postharvest quality. Nevertheless, correlation analysis does not establish a regulatory network or direct causal effects. Further transcriptomic, enzyme activity, targeted metabolomic, and metabolic flux analyses are needed to clarify the underlying mechanisms.

### 3.5. Microbial Community Analysis

**Figure 9:**
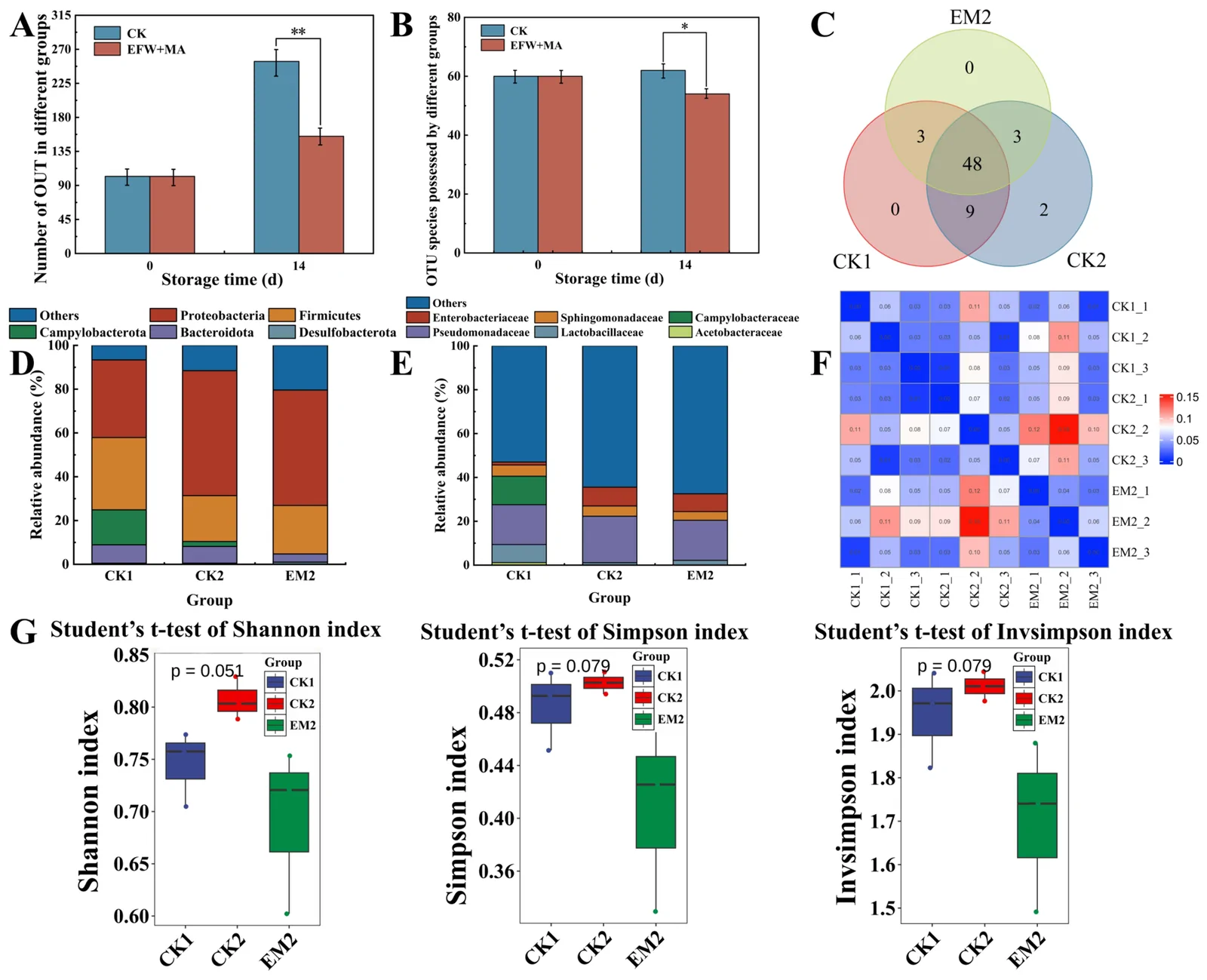
Effects of EFW+MA treatment on bacterial community composition and α-diversity of arrow bamboo shoots at day 14 of storage: (**A**) number of OTUs in different groups; (**B**) OTU categories identified in different groups; (**C**) Venn diagram showing OTU similarity among different treatments; (**D**) stacked bar chart of the relative abundance of bacterial taxa at the phylum level; (**E**) stacked bar chart of the relative abundance of bacterial taxa at the family level; (**F**) heatmap of Pearson correlation coefficients among samples; (**G**) boxplots of Shannon, Simpson, and InvSimpson diversity indices. * *p* < 0.05, ** *p* < 0.01.

Because bamboo shoots have high moisture content and are rich in sugars and various nutrients, they readily provide a suitable environment for microbial growth [37]. As shown in Figure 9A, the number of OTUs in the fresh sample CK1 was relatively low at 0 d of storage. After 14 d of storage, the number of OTUs in the CK2 group increased markedly to about 254, whereas that in the EM2 group was only about 155, indicating that this treatment significantly suppressed the increase in microbial abundance during storage. Figure 9B further shows that the number of identifiable species in the CK group increased from 60 to about 62, whereas it decreased to about 54 in the EFW+MA group, suggesting that EFW+MA not only reduced OTU richness but also decreased the number of identifiable species. Previous studies have shown that electrolyzed functional water can exert antimicrobial effects by inducing microbial DNA damage or interfering with microbial metabolic processes [3] and can reduce the risks of surface microorganisms and pesticide residues on fruits and vegetables while maintaining postharvest quality [28]. Therefore, the combined treatment significantly inhibited microbial proliferation and delayed spoilage during storage.

The Venn diagram results showed (Figure 9C) that CK1, CK2, and EM2 shared 48 OTUs, indicating a certain similarity in microbial composition among the different samples, which is generally consistent with the report by Xu et al. [35] on the microbial community composition of bamboo shoots. Meanwhile, CK2 had 2 unique OTUs, whereas no unique OTUs were detected in EM2, indicating that EFW+MA reduced the detection of treatment-specific OTUs at the later stage of storage. Analysis at the phylum level showed (Figure 9D) that the dominant bacterial phyla in bamboo shoot samples were mainly *Proteobacteria* and *Firmicutes*, which are also commonly found in fruit and vegetable storage systems [38]. After 14 d of storage, the relative abundance of *Proteobacteria* was higher in the EFW+MA group, whereas the relative abundances of *Firmicutes* and *Campylobacterota* were lower. In particular, *Campylobacterota* accounted for 2.261% in CK2 but was not detected in EM2. Given that *Campylobacterota* includes some common foodborne pathogenic bacteria and can improve environmental adaptability through biofilm formation [39], EFW+MA treatment may help reduce the risk of potential pathogenic contamination.

Results at the family level further showed that EFW+MA altered the microbial community structure of bamboo shoots (Figure 9E). After 14 days of storage, *Pseudomonadaceae* and *Enterobacteriaceae* were the main dominant bacterial families, followed by *Sphingomonadaceae*, which is consistent with the 16S rRNA-based analysis by Parlapani et al. [40], who found that *Pseudomonas* dominated spoilage-associated communities. Compared with CK2, the relative abundances of several bacterial families differed in EM2. Notably, the relative abundance of *Lactobacillaceae* increased from 1.049% in CK2 to 2.055% in EM2. Some lactic acid bacteria, including certain members of *Lactobacillaceae*, have been reported to exhibit bioprotective potential in food systems through the production of organic acids, bacteriocins, hydrogen peroxide, or other antimicrobial metabolites [41]. However, these properties are species- and strain-dependent. Because the present 16S rRNA amplicon sequencing data do not provide species- or strain-level identification, functional activity, or direct evidence of antimicrobial effects, the increase in *Lactobacillaceae* is interpreted only as a treatment-associated shift in bacterial community composition. Further studies involving species- or strain-resolved identification, microbial isolation, and functional validation are required to determine whether this taxon contributes to the preservation of arrow bamboo shoots.

Alpha-diversity analysis showed that the Shannon, Simpson, and Invsimpson indices were higher in CK2 than in CK1 but lower in EM2 than in CK2 (Figure 9G), indicating that EFW+MA was associated with limited expansion of bacterial diversity during storage. The β-diversity heatmap further showed clear separation between CK2 and EM2 (Figure 9F), confirming that the treatment was associated with a distinct bacterial community structure. Together with the lower OTU number and species richness in EM2, these community-level changes were consistent with the lower decay incidence and better postharvest quality of treated shoots [42]. However, 16S rRNA amplicon sequencing provides relative taxonomic information and cannot determine absolute microbial load, viability, or the direct functional contribution of individual taxa to spoilage.

## 4. Conclusions

Building on the superior preservation performance of EFW+MA established in our previous four-treatment study, the present study demonstrated that EFW+MA effectively delayed postharvest quality deterioration of arrow bamboo shoots during cold storage and provided integrated physiological, metabolomic, and microbial evidence for its preservation effect. The previous comparison also showed that MA was more effective than EFW when applied individually, indicating that the two methods did not contribute equally to preservation. Nevertheless, the combined treatment achieved the best overall preservation performance, suggesting that EFW and MA exerted complementary effects. EFW+MA created a low-O_2_/high-CO_2_ microenvironment inside the package and significantly reduced respiration intensity, weight loss, decay incidence, surface yellowing, and browning. At day 35, EFW+MA reduced weight loss and decay incidence by 78.7% and 82.0%, respectively, compared with the control.

Notably, EFW+MA suppressed postharvest tissue hardening and lignification, as reflected by reductions in lignin and cellulose contents of 34.6% and 22.9%, respectively, together with lower PAL, POD, and PPO activities. The treatment also enhanced antioxidant defense, increasing SOD, CAT, and APX activities by 62.6%, 20.9%, and 11.1%, respectively, while reducing MDA accumulation by 21.0%. Metabolomic analysis further showed that EFW+MA moderated storage-associated changes in organic acids, amino acids and their derivatives, phenolic acids, and lipids, particularly those related to phenylalanine metabolism, alpha-linolenic acid metabolism, and secondary metabolite biosynthesis. In parallel, EFW+MA reduced OTU number and species richness and altered the bacterial community structure.

Overall, EFW+MA extended the cold-storage shelf life of arrow bamboo shoots to 35 d, highlighting its practical potential as a residue-free preservation strategy. The combined physiological, metabolomic, and microbial results suggest that its preservation effect is associated with delayed lignification, improved oxidative-status maintenance, moderated storage-related metabolic changes, and altered bacterial-community composition. However, the metabolomic and 16S rRNA sequencing data identify treatment-associated changes rather than direct causal relationships. Future studies using targeted metabolite quantification, molecular analysis, and microbial functional validation will help clarify the mechanisms underlying EFW+MA-mediated quality preservation.

## Data Availability

The original contributions presented in this study are included in the article. Further inquiries can be directed to the corresponding author.
